# Sex-specific photosynthetic capacity and Na^+^ homeostasis in *Populus euphratica* exposed to NaCl stress and AMF inoculation

**DOI:** 10.3389/fpls.2022.1066954

**Published:** 2022-11-28

**Authors:** Na Wu, Zhen Li, Fei Wu, Lina Zhen

**Affiliations:** ^1^ Institute of Applied Biotechnology, College of Agriculture and Life Science, Shanxi Datong University, Datong, Shanxi, China; ^2^ Key Laboratory of State Forestry and Grassland Administration on Graphene Forestry Application, Shanxi Datong University, Datong, Shanxi, China; ^3^ College of Life Sciences, Northwest Normal University, Lanzhou, China

**Keywords:** Na^+^ homeostasis, photosynthesis, mycorrhiza, poplar, dioecious

## Abstract

Soil salinity and associated land degradation are major ecological problems. Excess Na^+^ ions in soil impede the plant photosynthetic process and Na^+^ homeostasis status. Arbuscular mycorrhizal fungi (AMF) can alleviate salt stress in host plants. Although a number of studies have demonstrated that Na^+^ accumulation is decreased by mycorrhizae, the molecular mechanisms involved have received little attention from researchers. *Populus euphratica* is a typical natural woody tree with excellent salt tolerance. Due to its symbiosis forming capability with AMF, we explored the influence of *Funneliformis mosseae* on the growth, photosynthesis, and expression of three genes involved in Na^+^ homeostasis within dioecious *P. euphratica* under salt stress. The results indicated that salt stress significantly increases Na^+^ contents and inhibits growth status and photosynthetic capacity, especially in females. However, AMF had positive effects on the growth status, photosynthetic capacity and Na^+^ homeostasis, especially in males. The expression levels of *NHX1* in shoots and *HKT1* and *SOS1* in roots, all of which are involved in Na^+^ homeostasis, were upregulated by *F. mosseae* under salt stress. For males, the beneficial effect of AMF centered on extruding, sequestering and long-distance transporting of Na^+^ ions . For females, the beneficial effect of AMF centered on extruding excessive Na^+^.

## Introduction

Salt stress affects more than 6% of land throughout the world and limits plant growth (Chen et al., 2014). The trait of salt-tolerance in woody plants involves multiple mechanisms at physiological and molecular levels that respond to soil salinity (Ruiz-Lozano et al., 2012). Under salt stress, Na^+^ homeostasis is a key physiological mechanism in plants, and the disruption of Na^+^ homeostasis at the whole-plant level could eventually result in the inhibition of photosynthesis capacity and growth (Porcel et al., 2016; Zhang et al., 2017). For most plants, Na^+^ is the main toxic ion, thus plants have adopted various kinds of strategies to avoid Na^+^ toxicity, such as extruding Na^+^ out of cell and sequestering Na^+^ into vacuoles (Porcel et al., 2016; Chen et al., 2017; Zhang et al., 2017). The cooperation of several Na^+^ transporters, such as the plasma membrane Na^+^/H^+^ antiporter SOS1, the Na^+^ transporter HKT1 and the tonoplast Na^+^/H^+^ antiporter NHX1, plays a key role in maintaining Na^+^ homeostasis status (Zhang et al., 2017). Previous studies have demonstrated that the SOS1 antiporter mainly mediates the loading of Na^+^ from xylem parenchyma cells (XPCs) into xylem in plant roots (Qi and Spalding, 2004), HKT transporters are responsible for unloading Na^+^ from xylem into XPCs in roots (Platten et al., 2006), and the NHX1 antiporter sequesters Na^+^ into vacuoles (Shi and Zhu, 2002).

Dioecious plants are essential components of terrestrial ecosystems, accounting for approximately 7.5% of angiosperm genera (Renner and Ricklefs, 1995). Male and female plants experience different selective pressures and respond differently to salt and other environmental stressors (Melnikova et al., 2017). Previous investigations have reported that males are less sensitive to abiotic stressors than females, and male-biased sex ratios are often found under abiotic stressors (Han et al., 2013). Chen et al. (2011) also found that male poplar cuttings had lower photosynthetic protein degradation, a higher expression of related genes and more protective systems than females under salt stress. Thus, the sexual dimorphism in physiological and molecular metabolism can change the sex ratios of dioecious plants in saline areas (Chen et al., 2011).

Most terrestrial plants can form symbioses with arbuscular mycorrhizal fungi (AMF) (Smith and Smith, 2011), which can improve plant tolerance to salt stress (Porcel et al., 2016; Chen et al., 2017). AMF improves salt tolerance by facilitating water uptake (Chen et al., 2017), increasing photosynthesis abilities (Estrada et al., 2013a) and maintaining ionic homeostasis (Estrada et al., 2013b). As a barrier for Na^+^ selection, AMF may be involved in the molecular mechanisms within plants under salt stress, such as the relative expression of *SOS1*, *HKT* or *NHX* genes related to Na^+^ ion homeostasis (Ruiz-Lozano et al., 2012; Chen et al., 2017). Interestingly, males and females of dioecious plants respond differently to AMF (Li et al., 2015). Vega-Frutis and Guevara (2009) reported a different colonization rate by AMF between male and female *Carica papaya*. Li et al. (2015) found that AMF had more positive effects on males than females under water stress. The different responses of the two sexes indicate that AMF may alter the sexual response to salt stress and influence the sex ratio and spatial distribution of males and females in saline environments. However, little is known about AM symbiosis in the molecular regulatory mechanisms of dioecious plants under salt stress.

*Populus euphratica*, a typical dioecious tree species, is widely distributed in saline areas in China and plays a crucial role in maintaining ecological balance and stability (Guo et al., 2021). Males are more frequent than females in saline areas, and females are listed as key plants protected by local governments in many regions (Han et al., 2013). By comparing the growth status, photosynthesis, and relative expression levels of key Na^+^ transporters involved in Na^+^ homeostasis in females and males exposed to AMF inoculation and soil salinity, this study elucidates the effects of AMF inoculation on the photosynthesis and Na^+^ homeostasis capacity of dioecious *P. euphratica* under salt stress and provide a theoretical basis about the effects of AMF as it pertains to maintaining the balance of dioecious plants in saline ecosystems.

## Materials and methods

### AMF inoculation

In this study, the AMF inoculum was *Funneliformis mosseae* Schenck & Smith (BGC XJ02), provided by Beijing Academy of Agriculture and Forestry Sciences, China. AMF inoculum was propagated with *Zea mays* and consisted of AM spores (100 per gram), mycelium, root fragments and soil.

### Plant and soil treatments

*Populus euphratica* plants (one year old) were collected from a plant nursery of the Sanggan River Experimental Bureau in Datong, Shanxi Province, China. Males and females were disinfected with 0.05% KMnO_4_ for 12 h and then rinsed three times by deionized water. The upper layer (5-20 cm) of the nursery poplar field was filtered through a 2 mm sieve to provide growth substrate for cuttings. Soil physio-chemical properties : pH (the soil and water ratio: 1:5) 7.9; soil organic carbon, 18.21 g·kg^-1^; available K, 44.82 g·kg^-1^; available P, 10.96 mg·kg^-1^; available N, 29.71 mg·kg^-1^ . The air- dried soil was sterilized by gamma rays.

### Experimental design

The experiment consisted of a completely randomized block design that included three factors: sex (male and female), AMF inoculation status (inoculated with sterile inoculum and *F. mosseae*) and salt stress (0 and 100 mM NaCl). There were 15 replications for each treatment, totaling 2×2×2×15 = 120 pots. Cuttings were grown in a greenhouse at 28°C. Sixty cuttings were separately inoculated with 50 g of AMF. The others were inoculated with 50 g of autoclaved AMF. After being maintained for 60 days, all pots were divided into two groups of 60 individuals, and each group was treated with different soil salinities. For salt stress, 60 pots were treated with 20 mM NaCl every 3 days and reached 100 mM 5 times. The control group was treated with sterilized water. Salt stress lasted for 25 days and subsequently the plants were harvested. For each treatment, six cuttings were selected to measure the AMF colonization rate, growth status, photosynthetic capacity parameters, Na^+^ contents and the relative expression of key Na^+^ transporters involved in Na^+^ homeostasis.

### AMF colonization rate

At harvest, the fresh roots of six randomly selected plants were immediately collected, gently washed, carefully cut into fragments (1 cm), and then fixed with by FAA solution. The solution contained 10% KOH and 0.05% trypan blue in lactophenol, which was used to stain root samples. AMF colonization status was determined under an optical microscope using the gridline intersection method (McGonigle et al., 1990). AMF colonization rates are shown as a percentage of colonized root length. Arbuscular mycorrhizal dependence was calculated using the following formula (Menge et al., 1978): Mycorrhizal dependency = (the dry weight of mycorrhizal cuttings/the dry weight of nonmycorrhizal cuttings) × 100%.

### Growth status

Stem length was measured by band tape, and ground diameter was measured with Vernier calipers at the beginning and end of the salt treatment. The relative growth rates of height and ground diameter were calculated using the following formula: Relative growth rate of height = [(final height – initial height)/initial height] × 100%; Relative growth rate of ground diameter = [(final ground diameter – initial ground diameter)/initial ground diameter] × 100%. At harvest, cuttings were placed at 105°C for 20 min in an oven to destroy enzyme activities, and then dried at 80°C to a constant weight to determine the accumulation of biomass.

### Photosynthetic capacity parameters

Net photosynthesis rates (Pn), stomatal conductance (Gs), intercellular CO_2_ concentration (Ci) and transpiration rates (E) were determined for six mature leaves from a three-hour period, using a Li-6400 portable photosynthesis system (Li-Cor Inc., Lincoln, NE, USA). Before chlorophyll fluorescence measures were made, fully expanded leaves from six randomly selected cuttings from each treatment were placed in the dark for 30 min at 25°C. The maximal fluorescence (Fm) and minimum fluorescence (Fo) yields were detected using a modulated chlorophyll fluorometer (Mini-Imaging-PAM, Walz, Germany). The maximum quantum yield (Fv/Fm) and actual quantum yield (ΦPSII) of photosystem II were calculated as follows: Fv/Fm = (Fm-Fo)/Fm; ΦPSII = (Fm’-Fs)/Fm’. The photochemical quenching (qP) and nonphotochemical quenching (NPQ) were calculated as follows: qP = (Fs-Fo’)/(Fm’-Fo’); NPQ = (Fm’-Fo’)/(Fm-Fo).

### Na^+^ determination

After drying at 80°C for 2 days, the shoot and root samples were ground to a homogeneous powder and passed through a 20 µm mesh screen. The dry powders were extracted with HCl solution overnight at 37°C. After centrifugation at 10,000 × g for 10 min, the supernatants were diluted and then analyzed using an atomic absorption spectrophotometer (Z-2000, Shimadzu, Japan) (Peng et al., 2004).

### Quantitative real-time PCR analysis

Shoots and roots were used to extract RNA with an RNeasy Plant Kit (R6827-01 50T Omega, USA). RNA was detected using a Nanodrop 1000 spectrophotometer (Nano-Drop Products, USA Thermo Fisher Scientific, USA). First-strand cDNA was synthesized by a first-strand cDNA synthesis kit (Tiangen Biotech, Peking, China). Transcripts of different treatments were determined using a CFX96 real-time PCR detection system (Bio-Rad, Hercules, CA, USA) and a Roche SYBR green system (Roche Diagnostics GmbH, SandhooferStraβe, Mannheim, Germany). The primers are listed in **Table 1**. Quantitative real-time polymerase chain reaction was conducted in a 20 μl reaction system, including 1 μl cDNA, 0.8 μl primer pairs (10 μM), 7.4 μl sterilized H_2_O and 10 μl SYBR Premix Ex Taq™ II. The PCR amplification of *PeSOS1* was performed using the following program: 4 min for denaturation at 94°C, 40 cycles of 20 s for denaturation at 94°C, 20 s for annealing at 55°C and 20 s for extension at 72°C. The PCR amplification of *PeHKT1* was performed using the following program: 1 min for denaturation at 95°C, 40 cycles of 20 s for denaturation at 94°C, 1 min for annealing at 60°C and 20 s for extension at 72°C. The PCR amplification of *PeNHX1* was performed with the following program: 5 min for denaturation at 94°C, 36 cycles of 40 s for denaturation at 94°C, 40 s for annealing at 56°C and 1 min for extension at 72°C. As an endogenous control, the ubiquitin gene (*UBQ*) was selected to normalize the relative expression levels of the three genes. Relative quantification was performed using the comparative 2^-ΔΔCT^ method (Livak and Schmittgen, 2001).

**Table 1:** Gene primers used for quantitative real-time PCR amplification.

Gene name	Primer-forward (5’-3’)	Primer-reverse (5’-3’)
*PeSOS1*	AAGGATCGGGGATGGAATTAG	GAAAAGAAGGGCAGGAAGGAA
*PeHKT1*	GCATCACAGAGAGGCGAAA	TCCATTTCCCTGAGAATCCA
*PeNHX1*	TTCGGTTTGAGGATGGTAT	AATGGCAAGGGCAGTAAT
*UBQ* (Couturier et al., 2007)	GTCCTCTTCCAGCCATCTC	TTCGGTCAGCAATACCAGG

### Statistical analysis

SPSS 22.0 software (SPSS Inc., Chicago, IL, USA) was used for the statistical analysis. Three-way analyses of variance (ANOVAs) were adapted to evaluate the significance of sex, AMF inoculation and salt stress and their interaction on cuttings at the significance level (*P* ≤ 0.05). The means were compared with Duncan’s multiple range test and LSD test. Figures were made using Sigmaplot 10.0 (SanJose, CA, United States).

## Results

### AMF colonization rate and mycorrhizal dependency

Cuttings inoculated with *F. mosseae* formed typical AMF structures, and cuttings without AMF inoculation did not form mycorrhizal structures. AMF colonization rates were over 75%, and no significant differences existed among different sexes and salt treatments. Salt stress significantly increased the mycorrhizal dependency of cuttings; however, female cuttings showed lower mycorrhizal dependency than male cuttings under the same salinity conditions (**Table 2**).

**Table 2:** Colonization rates and mycorhizal denpendence of *Populus euphratica* under different salt conditions.

Treatments	Colonization rate (%)	Mycorrhizal dependency
AM M 0 mM	80.21±3.11	106.14±6.92b
AM F 0 mM	79.12±4.33	94.21±5.15c
AM M 100 mM	77.64±4.02	121.26±5.66a
AM F 100 mM	78.47±3.75	113.26±5.05b

AM, AMF inoculation; NM, non-inoculation; M, Males; F, Females; 0 mM, without salt stress; 100 mM, under salt stress. Different letters indicate significant difference at P ≤ 0.05, the data are means ± SD (n = 6).

### Growth status

Males and females grown without salt stress showed a similar growth index and biomass accumulation (**Table 3**). Cuttings under salt stress showed declines in the relative growth rate of height, relative growth rate of ground diameter and dry weight. Compared to females, the relative growth rate of height, relative growth rate of ground diameter and dry weight were significantly higher in males under salt stress. In addition, males and females differed in their response to AM symbiosis. For males, the relative growth rate of height, relative growth rate of ground diameter and dry weight inoculated with AMF were 25.41%, 27.27% and 21.26% higher, respectively, than those of noninoculated ones under salt stress, whereas they were 8.43% lower, and 10.53% and 13.36% higher, respectively, in female cuttings. Two-way ANOVAs indicated that the relative growth rate of height and dry weight of male cuttings were significantly affected by the salt × sex interaction. Three-way ANOVAs indicated that relative growth rate of height and dry weight were significantly affected by sex and the salt × sex interaction in both sexes. The relative growth rate of height was also significantly affected by the interaction of AMF × sex.

**Table 3:** Effects of AMF inoculation on growth status of *P. euphratica* females and males under different salt conditions.

Sex	AMF inoculation	Salt (mM)	Relative growth rate of height (%)	Relative growth rate of ground diameter (%)	Dry weight (g)
Male	AM	0	3.91±0.33a	0.47±0.05a	19.72±1.33a
	NM	0	3.74±0.37a	0.43±0.07a	18.58±1.42a
	AM	100	2.27±0.25b	0.28±0.05b	11.35±1.14b
	NM	100	1.81±0.26c	0.22±0.06bc	9.36±0.97c
*P*_salt_			**	**	**
*P*_AMF_			*	NS	*
*P*_salt × AMF_			*	NS	*
Female	AM	0	3.65±0.37A	0.41±0.06a	19.02±1.41A
	NM	0	3.93±0.28A	0.46±0.04a	20.19±1.26A
	AM	100	1.52±0.33B	0.21±0.07b	9.14±1.23B
	NM	100	1.66±0.22B	0.19±0.05b	8.07±0.88B
*P*_salt_			**	**	**
*P*_AMF_			NS	NS	NS
*P*_salt × AMF_			NS	NS	NS
*P*_sex_			*	NS	*
*P*_salt × sex_			*	NS	*
*P*_AMF × sex_			*	NS	NS
*P*_salt × AMF × sex_			NS	NS	NS

AM, AMF inoculation; NM, non-inoculation; **: significant effect at P ≤ 0.01; *: significant effect at 0.01 ≤ P ≤ 0.05; NS, no significant effect P > 0.05. Different letters (lowercases for males and capital letters for females) indicate significant difference at P ≤ 0.05, the data are means ± SD (n = 6).

### Gas exchange parameters

**Figure 1:**
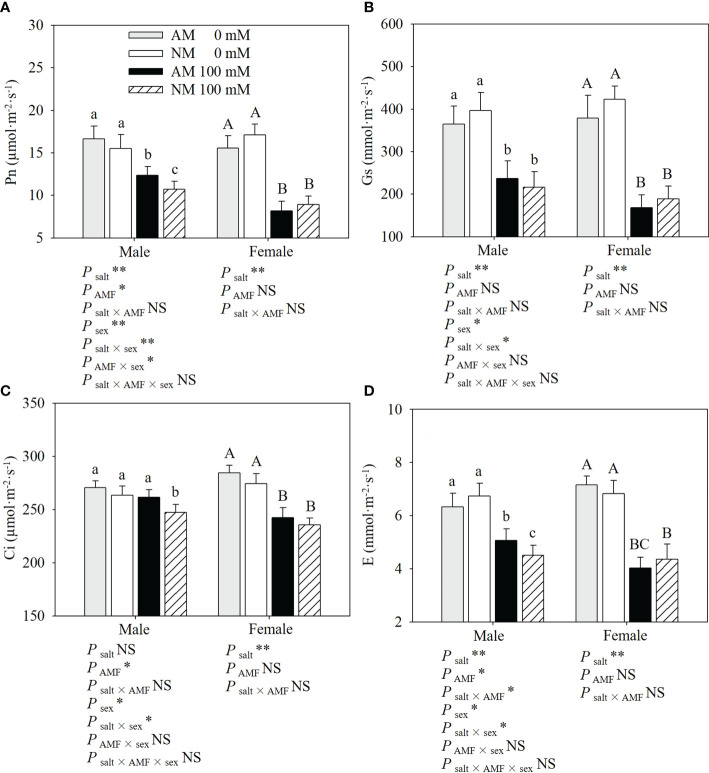
Effects of AMF inoculation on Pn **(A)**, Gs **(B)**, Ci **(C)** and E **(D)** of *P. euphratica* males and females under different salt conditions AM, AMF inoculation; NM, non-inoculation; 0 mM, without salt stress; 100 mM, under salt stress; AMF, AMF formation; **: significant effect at *P* ≤ 0.01; *: significant effect at 0.01 < *P* ≤ 0.05; NS, no significant effect. Different letters (lowercases for males and capital letters for females) indicate significant difference at *P* ≤ 0.05, the data are means ± SD (n = 6).

As shown in **Figure 1**, salt stress significantly reduced gas exchange parameters in both sexes, especially in nonmycorrhzal females. Under salt stress, male cuttings exhibited a higher Pn, Gs and Ci than females, and AMF inoculation showed positive effects on the gas exchange parameters in male cuttings. Compared with noninoculated cuttings, inoculated males showed a higher Pn (15.39%), Gs (9.53%), Ci (5.71%) and E (12.42%), while inoculated females showed a lower Pn (8.61%), Gs (10.98%), E (7.59%) and higher Ci (2.76%) under salt stress. AMF inoculation had more positive effects on male cuttings than on female cuttings. Two-way ANOVAs showed that for female cuttings, gas exchange parameters were significantly affected by salt stress, whereas for male cuttings, Pn, Gs and E were significantly affected by salt stress and Pn, Ci and E were significantly affected by AMF inoculation. The salt × AMF interaction only significantly affected E for male cuttings. Three-way ANOVAs indicated that sex and the interaction of salt × sex significantly affected gas exchange parameters. The interaction of sex × AMF only significantly affected Pn for male cuttings.

### Chlorophyll fluorescence parameters

**Figure 2:**
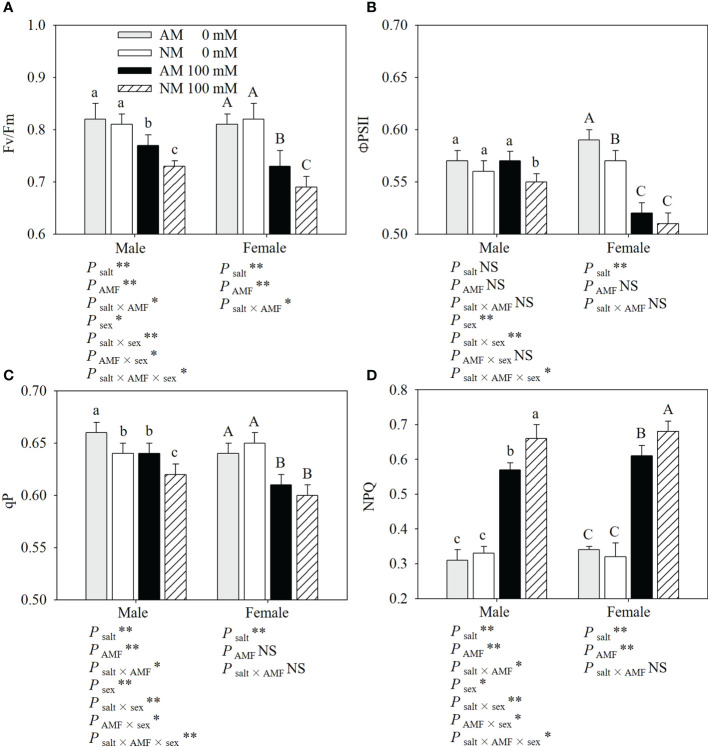
Effects of AMF inoculation on Fv/Fm **(A)**, ΦPSII **(B)**, qP **(C)** and NPQ **(D)** of *P. euphratica* males and females under different salt conditions AM, AMF inoculation; NM, non-inoculation; 0 mM, without salt stress; 100 mM, under salt stress; AMF, AMF formation; **: significant effect at *P* ≤ 0.01; *: significant effect at 0.01 < *P* ≤ 0.05; NS: no significant effect. Different letters (lowercases for males and capital letters for females) indicate significant difference at *P* ≤ 0.05, the data are means ± SD (n = 6).

Salt stress significantly reduced the Fv/Fm, ΦPSII and qP, and increased the NPQ of cuttings, especially in nonmycorrhizal females (**Figure 2**). Male cuttings under salt stress exhibited a higher Fv/Fm, ΦPSII and qP than female cuttings. Under salt stress, AMF showed positive effects on the chlorophyll fluorescence parameters in *P. cathayana*. Compared with noninoculated cuttings, inoculated males showed a higher Fv/Fm (5.48%), ΦPSII (3.64%), qP (3.23%) and lower NPQ (13.64%), while inoculated females showed a Fv/Fm (5.80%), ΦPSII (1.96%), qP (1.67%) and lower NPQ (10.29%) when subjected to salt stress. AMF inoculation had more positive effects on male cuttings than on female cuttings, which was mainly reflected in NPQ. Two-ANOVAs showed that except for ΦPSII in male cuttings, the other chlorophyll fluorescence parameters were significantly affected by salt. With the exception of ΦPSII and qP in females, AMF inoculation significantly affected the other chlorophyll fluorescence parameters. Three-ANOVAs indicated that chlorophyll fluorescence parameters had obvious differences between sexes, and were significantly affected by the salt × sex and salt × AMF × sex interaction.

### Na^+^ contents

**Figure 3:**
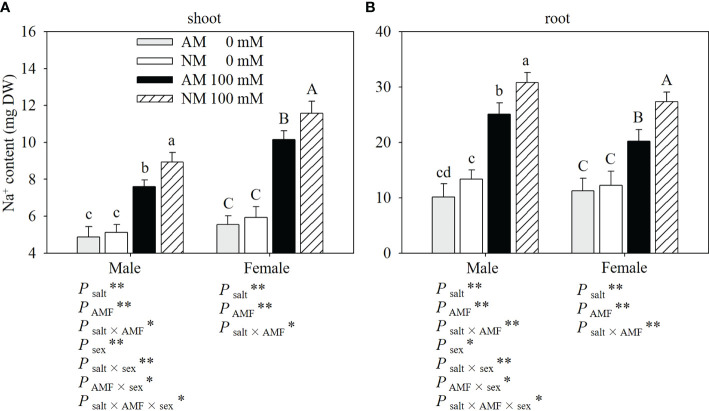
Effects of AMF inoculation on Na^+^ contents of shoots **(A)** and roots **(B)** in males and females under different salt conditions AM, AMF inoculation; NM, non-inoculation; 0 mM, without salt stress; 100 mM, under salt stress; AMF, AMF formation; **: significant effect at *P* ≤ 0.01; *: significant effect at 0.01 < *P* ≤ 0.05; NS: no significant effect. Different letters (lowercases for males and capital letters for females) indicate significant difference at *P* ≤ 0.05, the data are means ± SD (n = 6).

Salt stress increased the Na^+^ contents in the shoots and roots of plants (**Figure 3**). Mycorrhizal cuttings had significantly lower Na^+^ levels in shoots and roots than nonmycorrhizal cuttings under salt stress. When 100 Mm NaCl was applied, males showed higher Na^+^ levels in roots than females with 100 mM NaCl application. And for Na^+^ contents in shoots, males showed lower Na^+^ levels than females and lower Na+ levels in shoots than females. Two-ways ANOVAs showed that the Na^+^ contents in the shoots and roots were significantly affected by salt, AMF and salt × AMF interaction. Three-way ANOVAs indicated that Na^+^ contents in shoots and roots were significantly affected by sex and the interaction of salt × sex, AMF × sex and salt × AMF × sex.

### Expression patterns of *PeSOS1*, *PeHKT1* and *PeNHX1*

**Figure 4:**
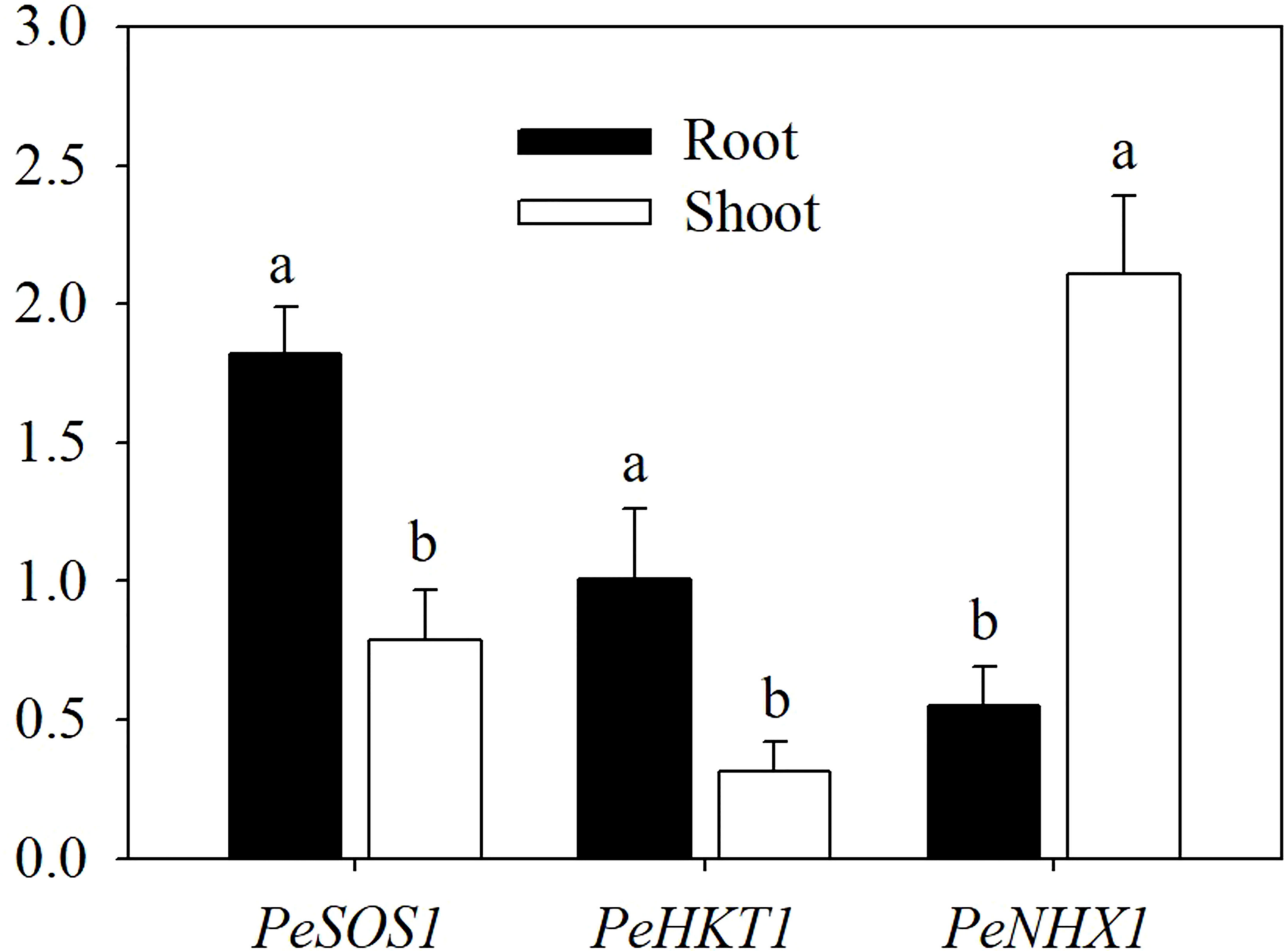
Tissue-specific expression of *PeSOS1*, *PeHKT1* and *PeNHX1* under salt stress. Different letters indicate significant difference at P ≤ 0.05, the data are means ± SD (n = 6).

**Figure 5:**
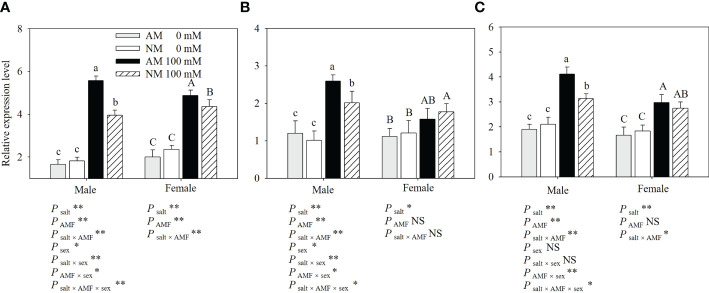
Effects of AMF inoculation on relative expression level of *PeSOS1* **(A)**, *PeHKT1* **(B)** and *PeNHX1* **(C)** of *P. euphratica* males and females under different salt conditions AM, AMF inoculation; NM, non-inoculation; 0 mM, without salt stress; 100 mM, under salt stress; AMF, AMF formation; **: significant effect at *P* ≤ 0.01; *: significant effect at 0.01 < *P* ≤ 0.05; NS: no significant effect. Different letters (lowercases for males and capital letters for females) indicate significant difference at *P* ≤ 0.05, the data are means ± SD (n = 6).

To investigate the tissue-specific expression of *PeSOS1*, *PeHKT1* and *PeNHX1* in *Populus euphratica*, qRT−PCR was performed to assess the relative expression of the three genes. The results showed that *PeSOS1* and *PeHKT1* were expressed mainly in roots, while *PeNHX1* was expressed mainly in shoots (**Figure 4**). Here, we analyzed the relative expression levels of *PeSOS1* and *PeHKT1* in roots and *PeNHX1* in shoots. Irrespective of AMF inoculation, salt stress upregulated the relative expression levels of *PeNHX1* in shoots and *PeSOS1* and *PeHKT1* in roots (**Figure 5**). The relative expression of *PeSOS1* in roots showed a rapid and continuous increase under salt stress and was 2.18- fold higher in males and 1.85- fold higher in females than under control conditions. The expression of *PeHKT1* in male and female roots increased by 1.99- fold and 1.46-fold, respectively, under salt stress as compared to the control. *PeNHX1* in male and female shoots was up-regulated by 1.48- fold and 1.42-fold, respectively, under 100 mM NaCl compared to the control. The symbiosis of AMF upregulated the relative expression levels of the three genes under salt stress. Under application with 100 mM NaCl, the expression levels of *PeSOS1*, *PeHKT1* and *PeNHX1* in male cuttings that received AMF inoculation were 40.66%, 28.86% and 31.63% higher than those of noninoculated cuttings. The expression levels of *PeSOS1* and *PeHKT1* in female cuttings that received AMF inoculation were 11.67% and 8.36% higher than those in non-inoculated cuttings. Two-ANOVAs indicated that the relative expression of *PeSOS1*, *PeHKT1* and *PeNHX1* was significantly affected by salt stress. It also showed that the relative expression of the three genes in males and the relative expression level of *PeSOS1* in females were significantly affected by AMF inoculation and the salt × AMF interaction. Three-ANOVAs indicated that the relative expression levels of *PeSOS1* and *PeHKT1* were significantly affected by sex and the salt × sex interaction. The relative expression of the three genes was significantly affected by the AMF × sex and salt × AMF × sex interaction.

## Discussion

Salt stress could affect plants at the physiological and molecular levels, thus severely limiting plant growth status and biomass accumulation (Porcel et al., 2016). Female cuttings were more sensitive to salt stress, and AMF colonization may alleviate salt stress at the physiological and molecular levels by enhancing photosynthesis capacity and modulating Na^+^ homeostasis, which, according to the present study, decreased damage caused by salt stress, especially for males. In this study, *P. euphratica* showed a high AMF colonization rate, illustrating that poplar is a suitable species hosting plants (Liu et al., 2014; Wu et al., 2016).

Growth status is the most obvious trait reflecting plant development when subjected to AMF inoculation and abiotic stress (Evelin et al., 2009; Porcel et al., 2016). In the present study, although the growth index and biomass accumulation of cuttings were inhibited by salt stress, mycorrhizal cuttings grew better than nonmycorrhizal cuttings under salt stress, especially for males, suggesting AMF mitigated salt stress in dioecious poplar. Under salt stress, the beneficial influence of AMF inoculation on the growth index and biomass accumulation in plants was also found in other plants, including *P. cathayana*, *Euonymus maackii* and *Robinia pseudoacacia*. (Wu et al., 2016; Chen et al., 2017; Li et al., 2020).

The reduction in plant growth under salt stress can result from its inhibition of photosynthetic capacity (Porcel et al., 2015). In this study, salt stress decreased the gas exchange parameters of *P. euphratica*, which was consistent with previous studies (Wu et al., 2016; Chen et al., 2017; Li et al., 2020). Pn, Gs and Ci decreased less in males than in females under salt stress, which is in agreement with other observations (Chen et al., 2010; Wu et al., 2015). Our results indicate that AMF inoculation has a positive impact on Pn, Ci and E in males under salt stress. Chlorophyll fluorescence parameters can provide further insights into the photosynthetic apparatus damaged by stressors (Li et al., 2015; Porcel et al., 2015). According to results, the photosynthetic apparatus was damaged under salt stress,while females suffered more damage to the electron transport chain under salt stress. AMF inoculation had a positive effect on the chlorophyll fluorescence parameters in males and Fv/Fm and NPQ in females under salt stress, implying that mycorrhizal plants had a higher PSII efficiency under salt stress, especially in males.

As documented extensively, Na^+^ ions are the main toxic ion for most plants, therefore it is essential to maintain lower Na^+^ contents within plants (Evelin et al., 2009). Previous researches have reported that many plants, including *P. cathayana* (Wu et al., 2015), *Robinia pseudoacacia* (Chen et al., 2017) and *Euonymus maackii* (Li et al., 2020), can effectively prevent Na^+^ uptake under NaCl stress. Janz et al. (2012) found that salt-sensitive plants inhibited Na^+^ transport less efficiently than salt-tolerant plants. In our study, Na^+^ contents in the male shoots were significantly lower than those in females, and in the roots of male cuttings, Na^+^ contents were significantly higher than those in females, showing an efficient inhibition of Na^+^ transport in male cuttings. Moreover, mycorrhizal plants had a lower accumulation of Na^+^ within plants. Based on the comparatively improved performance of mycorrhizal plants as it pertains to the reduction of Na^+^ accumulation, we decided to investigate the response of three genes involved in the Na^+^ transport system.

The plasma membrane Na^+^/H^+^ transporter SOS1 plays a key role in the transport of Na^+^ and is expressed mainly in the roots of plants (Xu et al., 2008; Guo et al., 2012). Our results also showed that the relative expression level of *PeSOS1* in roots was strongly upregulated by NaCl application. In addition, Shi et al. (2002) found that the encoding gene *AtSOS1* is preferentially expressed in parenchyma cells, and is involved in loading Na^+^ into xylem to control Na^+^ delivery to the shoots . In another study, Ma et al. (2014) indicated that *ZxSOS1* was involved in the long-distance transport and spatial distribution pattern of Na^+^ ions. In the present study, the relative expression level of *PeSOS1* displayed a rapid and persistent increasing trend under 100 mM NaCl conditions, implying that *PeSOS1* plays a key role in the delivery of Na^+^ to shoots through loading Na^+^ into the xylem.

HKT proteins play key roles in regulating Na^+^ transport and maintaining Na^+^ homeostasis within plants (Horie et al., 2009). In general, the Na^+^ transporters HKT1 is encoded by the *HKT1* gene and mediates Na^+^ retrieval from xylem in plants (Byrt et al., 2007). *OsHKT1* mediates Na^+^ exclusion by removing Na^+^ from the xylem in roots, thus preventing Na^+^ over accumulation in shoots. Moreover, Sunarpi et al. (2005) reported that *AtHKT1* played a crucial role in protecting plants from Na^+^ toxicity, mainly focus on selectively unloading Na^+^ directly from xylem vessels to XPCs and reducing Na^+^ content in xylem . Our present results show that *PeHKT1* is mainly expressed in roots and is also upregulated by salt stress, implying that *PeHKT1* plays a significant role in unloading Na^+^ from xylem to parenchyma cells in roots. Therefore, we thought that Na^+^ accumulation in shoots would induce the expression of *PeHKT1*, thus facilitating excessive Na^+^ unloading into XPCs and consequently alleviating Na^+^ toxicity.

Sequestering Na^+^ into vacuoles is one of the crucial strategies for plants under salt stress (Chen et al., 2017). NHX1 plays a key role in compartmentalizing Na^+^ into vacuoles to maintain Na^+^ homeostasis (Shi and Zhu, 2002). Previous studies reported that the relative expression of *NHX1* in shoots was increased under salt stress (Zhang et al., 2017). Cosentino et al. (2010) found that the relative expression of *McNHX1* reached a high level in leaves under salt stress. Chen et al. (2017) found that *Robinia pseudoacacia RpNHX* was also preferentially expressed in leaves and was significantly induced by NaCl application. In our study, the expression level of *PeNHX1* in shoots was significantly upregulated by 100 mM NaCl, indicating that *PeNHX1* sequestraters Na^+^ in shoots.

The symbiosis of AMF significantly enhanced the relative expression of the three genes under salt stress, especially in males, thus providing a better understanding of the regulation of Na^+^ homeostasis by controlling Na^+^ transport systems in mycorrhizal-woody poplar. For males, the upregulation of the three genes by AM symbiosis showed that AMF plays a key role in extruding Na^+^ out of cell, sequestering Na^+^ into vacuoles and controlling Na^+^ long-distance transport. For females, the upregulation of *PeSOS1* in roots by AM symbiosis may promote Na^+^ export back to the soil and reduce the influx of Na^+^ into roots (Chen et al., 2017).

## Conclusion

**Figure 6:**
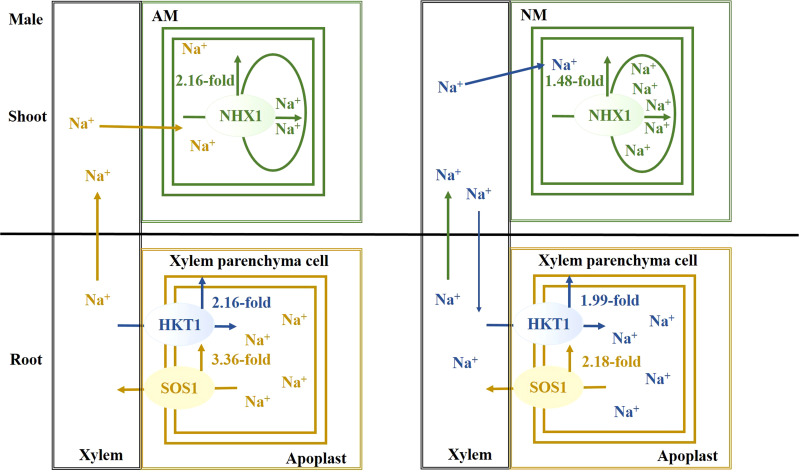
The schematic model for Na^+^ transporters regulating Na^+^ homeostasis for males.

**Figure 7:**
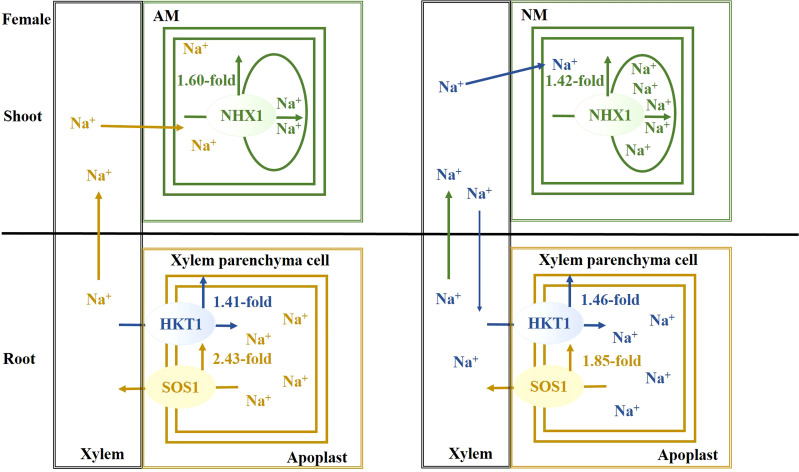
The schematic model for Na^+^ transporters regulating Na^+^ homeostasis for females.

Salt stress causes a disruption of Na^+^ homeostasis, accompanied by attenuation of photosynthetic capacity, thereby resulting in the inhibition of plant growth and biomass accumulation. Females are more sensitive to salt stress. When excessive Na^+^ exists, a mycorrhizal association facilitates Na^+^ secretion from root, Na^+^ unloading from xylem and Na^+^ compartmentalization into vacuoles, especially in males. As a result of AMF inoculation, there is an upregulation of *PeNHX1* expression in shoots and *PeSOS1* and *PeHKT1* in roots, when compared with nonmycorrhizal plants under salt stress. The beneficial effect of AMF centered on extruding, sequestering and long-distance transporting of Na+ ions for males (**Figure 6**), while the beneficial effect of AMF centered on extruding excessive Na+ for females (**Figure 7**). In summation, this study reveals that the beneficial effects of AMF on Na^+^ homeostasis are demonstrated by enhanced photosynthetic capacity, growth performance and biomass accumulation under salt stress.

## Data availability statement

The original contributions presented in the study are included in the article/supplementary material. Further inquiries can be directed to the corresponding author.

## Author contributions

NW and ZL have contributed equally to this work. NW and ZL performed the experiment, analyzed the experimental data and wrote the paper. FW and LZ revised the manuscript. All authors contributed to the article and approved the submitted version.

## Funding

This study was supported by Basic Research Program of Shanxi Province (20210302124247), Scientific and Technological Innovation Project of Shanxi Province (2021L376 and 2021L373), and the National Natural Science Foundation of China (31901227 and 32001297).

## Conflict of interest

The authors declare that the research was conducted in the absence of any commercial or financial relationships that could be construed as a potential conflict of interest.

## Publisher’s note

All claims expressed in this article are solely those of the authors and do not necessarily represent those of their affiliated organizations, or those of the publisher, the editors and the reviewers. Any product that may be evaluated in this article, or claim that may be made by its manufacturer, is not guaranteed or endorsed by the publisher.
